# Case Report: Variable Pharmacokinetic Profile of Eculizumab in an aHUS Patient

**DOI:** 10.3389/fimmu.2020.612706

**Published:** 2021-01-15

**Authors:** Romy N. Bouwmeester, Mendy Ter Avest, Kioa L. Wijnsma, Caroline Duineveld, Rob ter Heine, Elena B. Volokhina, Lambertus P. W. J. Van Den Heuvel, Jack F. M. Wetzels, Nicole C. A. J. van de Kar

**Affiliations:** ^1^ Department of Pediatric Nephrology, Radboud Institute for Molecular Life Sciences, Amalia Children’s Hospital, Radboud University Medical Center, Nijmegen, Netherlands; ^2^ Department of Pharmacy, Radboud University Medical Center, Nijmegen, Netherlands; ^3^ Department of Nephrology, Radboud Institute for Molecular Life Sciences, Radboud University Medical Center, Nijmegen, Netherlands

**Keywords:** atypical hemolytic uremic syndrome, aHUS, eculizumab, therapeutic drug monitoring, pharmacokinetic, proteinuria

## Abstract

**Background:**

With the introduction of eculizumab, a C5-inhibitor, morbidity and mortality improved significantly for patients with atypical hemolytic uremic syndrome (aHUS). In view of the high costs, actual needs of the drug, and increasing evidence in literature, aHUS patients can be treated according to a restrictive eculizumab regimen. We retrospectively analyzed the pharmacokinetic and dynamic parameters of eculizumab in one patient in time, emphasizing various factors which could be taken into account during tapering of treatment.

**Case Presentation:**

A nowadays 18-year-old male with a severe, frequently relapsing form of atypical HUS due to a hybrid CFH/CFHR1 gene in combination with the homozygous factor H haplotype, required chronic plasma therapy (PT), including periods with plasma infusion, from the age of onset at 5 months until initiation of eculizumab at the age of 11 years. A mild but stable chronic kidney disease (CKD) and 9 years of disease remission enabled prolongation of eculizumab interval. At the age of 15 years, a sudden yet multifactorial progression of chronic kidney disease (CKD) was observed, without any signs of disease recurrence. However, an acquired glomerulocystic disease, a reduced left kidney function, and abnormal abdominal venous system of unknown etiology were found. In addition, after an aHUS relapse, an unexpected increase in intra-patient variability of eculizumab concentrations was seen. Retrospective pharmacokinetic analysis revealed a change in eculizumab clearance, associated with a simultaneous increase in proteinuria.

**Conclusion:**

High intra-patient variability of eculizumab pharmacokinetics were observed over time, emphasizing the necessity for adequate and continuous therapeutic drug monitoring in aHUS patients. Eculizumab serum trough levels together with complement activation markers (CH50) should be frequently assessed, especially during tapering of drug therapy and/or changing clinical conditions in the patient. In addition, an increase in proteinuria could result in urinary eculizumab loss, indicating that urinary monitoring of eculizumab may be important in aHUS patients with an unexplained decline in serum concentrations.

## Introduction

The introduction of the complement therapeutic eculizumab led to a new era for patients with atypical hemolytic uremic syndrome (aHUS). aHUS is a rare and severe form of thrombotic microangiopathy (TMA) characterized by thrombocytopenia, microangiopathic hemolytic anemia (MAHA) and acute kidney failure (1). Dysregulation and overactivation of the alternative pathway (AP) of the complement system causes (glomerular) endothelial cell injury, ultimately leading to TMA (2). Currently, genetic mutations in complement factor and regulatory proteins are found in 50%–60% of patients with aHUS (3). The most frequent genetic alterations observed in aHUS are complement factor H (CFH) defects, including heterozygous mutations, or autoantibodies, all associated with a high risk of relapse and worse disease outcome (4–6). In 3%–5% of all aHUS cases, a genetic rearrangement between CFH and CFH related proteins (CFHR1-5) is found (7, 8). These CFH/CFHR hybrid proteins are associated with a structurally decreased complement regulatory function on the endothelial surface and a frequently relapsing form of aHUS (9, 10).

Before the implementation of complement therapeutics, plasma therapy (PT) was the only available treatment for patients with aHUS (11). In 2011, this changed dramatically with the introduction of eculizumab, the first monoclonal antibody targeting complement factor C5. By binding to C5, eculizumab prevents the splicing of C5 into C5a and C5b, and ultimately blocks the formation of the terminal membrane attack complex C5b-C9. With the introduction of eculizumab, morbidity and mortality improved significantly (12). The drug label advises a maintenance dose of 1200 mg biweekly in adult patients and pediatric patients with a body weight ≥40 kg. Treatment with eculizumab is very expensive, and dosing regimen as well as duration of treatment are topics of international debate. Moreover, multiple studies showed that it is safe and (cost-)efficient to taper and/or withdraw eculizumab therapy (6, 13, 14). During the maintenance phase, treatment with eculizumab at a reduced dose (through prolongation of the dose interval) while maintaining adequate complement blockade is now a common regimen in the Netherlands (6). Such personalized therapy is possible if therapeutic drug monitoring (TDM) is controlled by measuring eculizumab through levels and/or measuring of complement hemolytic activity (CH50) (15, 16). In aHUS, serum eculizumab concentrations of 50–100 µg/ml are recommended by the drug label for complete complement blockade (defined by CH50 <10%) (16, 17). Although various studies describe the utility of eculizumab TDM, adequate implementation in clinical care remains challenging. We retrospectively analyzed the pharmacokinetic and dynamic parameters of eculizumab in one patient during 4 years of eculizumab treatment, emphasizing various factors which could be taken into account during tapering of treatment.

## Case Presentation

**Figure 1:**
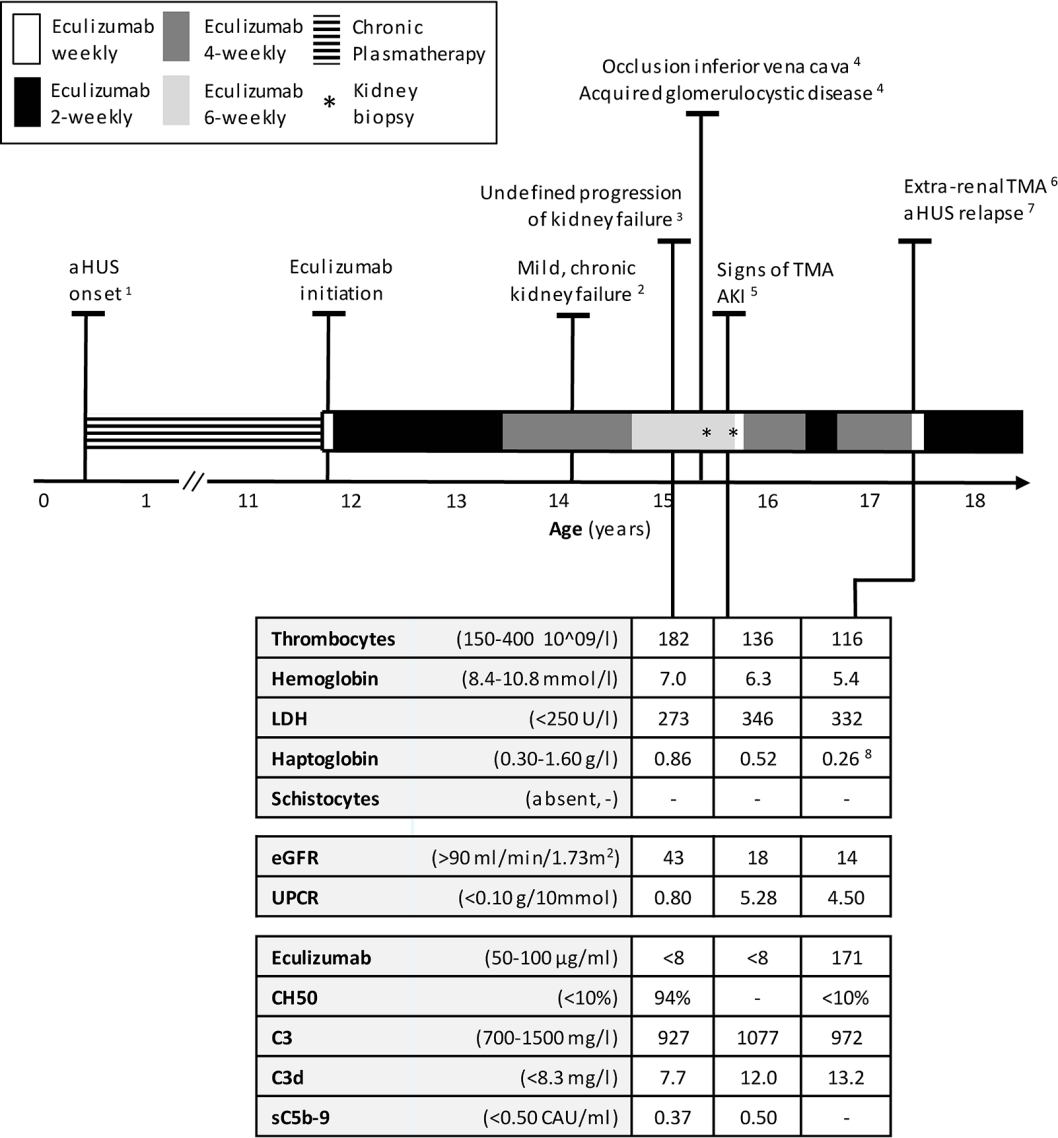
Timeline of treatment of aHUS in this patient, starting with the onset of disease at the age of 5 months until 18 years. Normal values of laboratory parameters indicated between parentheses. ^1^ After onset: chronic PT, including periods with plasma infusion. Every attempt to withdrawal in his first 4 years initiated a relapse. Number of relapses during PT period, n=7, ^2^ eGFR 65-70 ml/min/1.73m^2^, UPCR 0.18–0.53 g/10 mmol, ^3^ No thrombocytopenia, no signs of hemolysis; kidney biopsy right kidney: chronic TMA, no signs of interstitial nephritis or acute TMA,25% IFTA but diffuse small cysts eci, ^4^ Date of onset unknown, ^5^ AKI defined by a sudden increase in proteinuria and serum creatinine; kidney biopsy; similar to previous biopsy, ^6^ A traumatically obtained, extremely painful, and non-healing ulceration was considered as a (trigger of) an extra-renal manifestation of TMA, ^7^ aHUS relapse defined by AKI, thrombocytopenia and signs of hemolysis. ^8^ Haptoglobin decreased from 1.89 to 0,26 g/L. aHUS, atypical Hemolytic Uremic Syndrome; AKI, Acute Kidney Injury; eGFR, estimated Glomerular Filtration Rate (Schwartz formula); IFTA interstitial fibrosis and tubular atrophy; PT, Plasmatherapy; TMA, Thrombotic Microangiopathy; UPCR, Urine Protein-to-Creatinine Ratio.

Here we report a nowadays 18-year-old male with a severe, frequently relapsing form of aHUS. At the age of 5 months, our patient presented with TMA and nephrotic range proteinuria (**Figure 1**). Genomic analysis using MLPA (multiplex ligation-dependent probe amplification) identified a heterozygous deletion of CFH Exon 23, CFHR3 and CFHR1 Exon 1-5, indicating a genomic rearrangement resulting in a CFH/CFHR1 hybrid gene (NC_000001.10: g.(196715130_196716240)_(196799813_196800926)del); LRG_47:p.(Ser119Leu) and LRG_47:p.(Val1197Ala)). In addition, the at-risk CFH-H3 haplotype was found in homozygosity (18). Mutations in complement factor I and B (CFI, CFB), C3, membrane cofactor protein (MCP/CD46), thrombomodulin (THBD), and diacylglycerol kinase epsilon (DGKe) were excluded. Patient was negative for autoantibodies against CFH (detection performed using an in-house enzyme-linked immunosorbent assay (ELISA) (19).

Paternal family history was positive for aHUS. Our patient’s father is carrier of the same CFH/CFHR1 hybrid gene, however remained unaffected until present-day. Unfortunately, genetic analysis of the grandparents could not be performed, especially since two grandnieces of our patient developed aHUS over time: one fatal case more than 3 decades ago (age 32 years, no genetic analysis available) and one case, who presented with aHUS at the age of 47 years. Over time, she developed end stage kidney disease (ESKD) and recently presented with a first relapse after 14 years of disease remission. Genetic analysis confirmed a CFH/CFHR1 hybrid gene, identical to our patient’s and his father’s. The CFH-H3 risk haplotype was only found in heterozygous condition.

During follow-up of our patient, an unexpected yet unexplained chronic occlusion of the inferior vena cava (below renal veins) with substantial collateral formation was found. Furthermore, at the age of 15, imaging and biopsy revealed the remarkable presence of multiple, small medullary cysts, predominantly in the left kidney. Function of the left kidney was severely reduced (18%–28%) compared to the right kidney. Whole-exome sequencing (WES- kidney package, including cystic kidney diseases) did not provide any aberrations, and the etiology of the acquired glomerulocystic disease remained unknown. Only a few aHUS cases with an acquired cystic kidney disease (ACKD) of unknown (exact) pathogenesis have been described previously (20, 21).

Before the introduction of eculizumab, our patient required chronic PT. In his first four years, we attempted to withdraw PT on various occasions, yet this led to disease recurrence after (mostly infectious) triggering events. From the age of four until eleven years, disease remission was maintained with PT which, over the years, included plasma infusion (15–20 ml/kg) once weekly and plasma exchange (PE) once every 8^th^ week. At the age of eleven years, eculizumab was initiated and PT was stopped. After two years of additional remission, the eculizumab dosing interval was extended from 2 to 4 weeks. To monitor therapy next to TMA parameters, eculizumab concentrations and complement activity were regularly analyzed. Eculizumab detection was performed using an in‐house ELISA assay. Complement activity was accessed by determining the activity of classical complement route (CH50) using an ELISA method adapted from Roos et al. (22). Both methods have been described previously (15). During this 4 weekly dosing interval, eculizumab concentrations were adequate (70–84 µg/ml) and complement activity remained suppressed (CH50 <10%) (**Table 1**). Despite a mild, chronic kidney failure (eGFR 65–70 ml/min/1.73m^2^, urine protein-to-creatinine ratio (UPCR) 0.18–0.53 g/10mmol), overall clinical situation remained stable (**Figure 1**). In addition, antihypertensive medications effectively regulated blood pressure.

**Table 1:** Factors of potential influence to eculizumab clearance.

Age years normal value	Eculizumab serum 50–100 μg/ml	Eculizumab-C5 complex	C5 42–93 µg/ml	sC5b-9 <0.5 CAU/ml	Albumin serum 35-55 g/L	Total IgG serum7.0–16.0 g/L	Urinary PCR<0.10 g/10 mmol
***2-weekly interval*** *Mild, chronic kidney failure*							
**12.4**	598	199	38	0.37	34	8.67	0.27
**12.7**	558	234	87	0.55	34	9.52	0.28
***4-weekly interval*** *Mild, chronic kidney failure*							
**13.10**	70	–	84	0.35	36	10.72	0.54
**14.5**	84	188	93	0.25	39	8.51	0.79
***4-weekly interval*** *Signs of TMA, AKI*							
**16.1**	13	109	127	0.39	32	6.71	2.49
**16.3**	12	85	110	0.30	32	5.42	1.59
***2-weekly interval***							
**16.5**	127	196	109	0.63	31	5.09	2.63
**16.6**	167	229	103	0.39	34	4.90	3.43
***4-weekly interval***							
**16.10**	19	107	103	0.30	33	6.32	1.77
**17.1**	12	82	106	0.38	32	6.05	2.01

Relevant factors of potential influence to eculizumab clearance, i.e. eculizumab-C5, C5, sC5b-9, serum albumin levels, total serum IgG, and urinary protein to creatinine ratio measured in the different interval treatments of eculizumab. Target therapeutic ranges for eculizumab and normal reference ranges for C5, sC5b-9, albumin, total IgG, and urine protein-to-creatinine ratio (UPCR) are given.

### Undefined Progression of Kidney Failure

**Figure 2:**
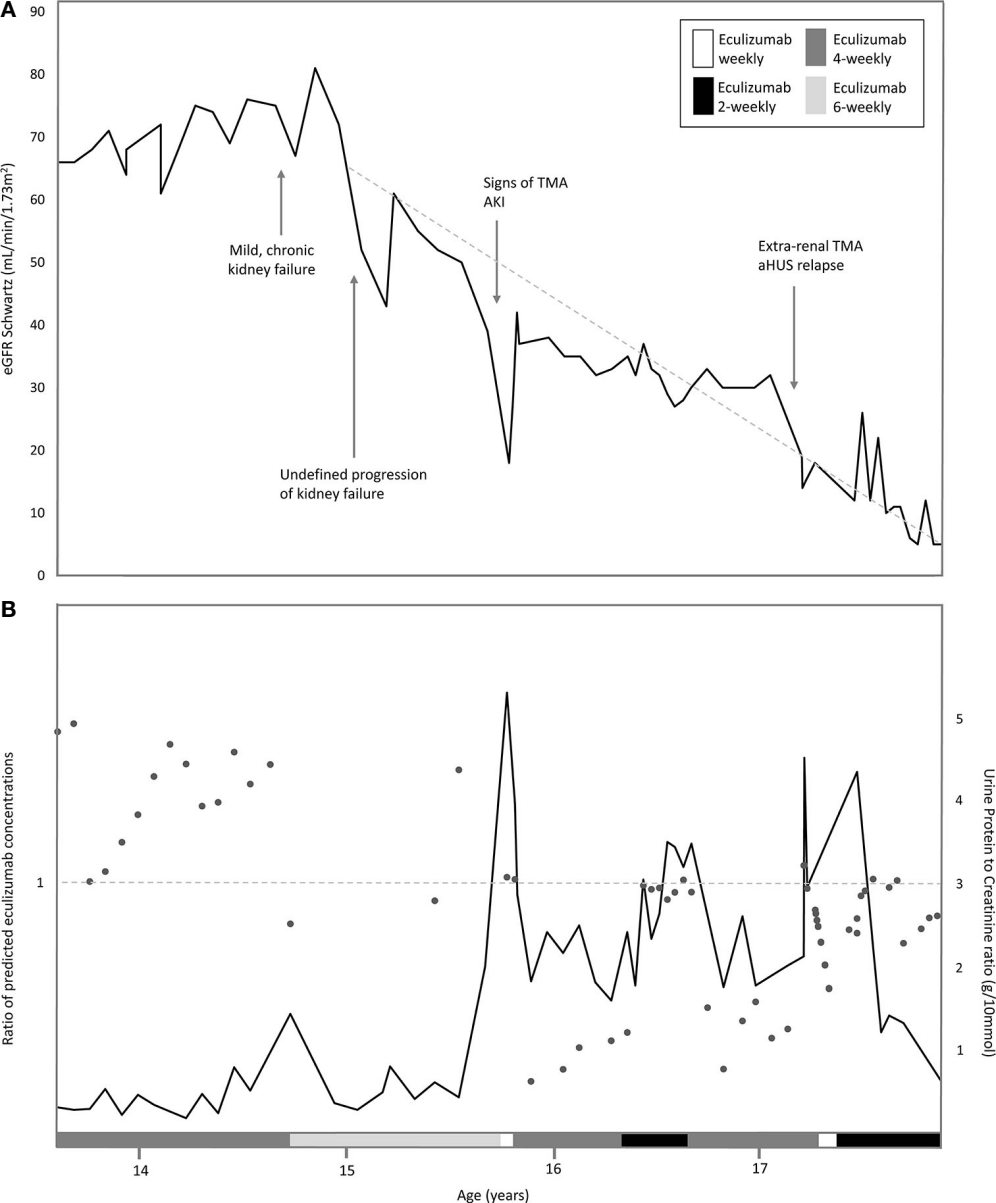
Overview of eGFR, proteinuria and ratio of predicted eculizumab concentrations related to the eculizumab treatment in time. **(A)** Estimated eGFR (Schwartz formula) indicated with dark grey line, and the various interval treatment periods of eculizumab, indicated by the legends: white bar: eculizumab 1200 mg weekly middle grey colored bar: four-weekly 1200 mg eculizumab; light-grey colored bar 6-weekly 1200 mg eculizumab; dark grey colored bar: two-weekly 1200 mg eculizumab. Dotted line indicates the annual eGFR decline of 22.9 ml/min/1.73m^2^ (baseline eGFR 68 ml/min/1.73m^2^). **(B)** Urine Protein-to-Creatine ratio (g/10 mmol) in time indicated with dark grey line. Ratios of predicted eculizumab concentrations of our patient and a standardized patient of same weight indicated with dots. Ratio =1 (horizontal dotted line), normal clearance; Ratio >1, decreased clearance in our patient (eculizumab concentrations higher than expected); Ratio <1; increased clearance in our patient (eculizumab concentrations lower than expected.

After three years of eculizumab therapy, the dosing interval was further extended to every 6 weeks. As before, the patient was carefully monitored and instructed when to seek medical attention. Eculizumab serum trough levels decreased to subtherapeutic and undetectable concentrations (<8–10 µg/ml). An unblocked terminal complement system (CH50 94%–149%) was now observed and accepted due to a very stable clinical situation and taken into account that the patient was stable for years with only once weekly plasma-infusion (which suggested that full complement blockade over the entire treatment interval might not be necessary). After six months, an unexplained decrease in kidney function was observed (eGFR 43 ml/min/1.73m^2^), with a mild increase in proteinuria (UPCR 0.80 g/10 mmol) (**Figure 2**). Hemoglobin-, thrombocytes, and haptoglobin levels were not decreased, LDH not elevated and schistocytes absent (**Figure 1**). In addition, blood pressure was normal (118/68 mmHg). Therefore, evident signs indicating aHUS disease recurrence were absent. Complement analysis showed no signs of (over)activation (C3 927 m g/L (normal range 700–1500), C3d 7.7 mg/L (<8.3), and sC5b-9 (0.37 CAU/ml (<0.50)). C3 levels were determined using ELISA, turbidometry (Cobas 8000 platform, F. Hoffman-La Roche Ltd, Basel, Switzerland) and C3d, sC5b-9, and C3bBbP levels by ELISA, as described before (23–25). While awaiting kidney biopsy in his best functioning right kidney, with the differential diagnosis of acute TMA or tubulointerstitial nephritis (TIN), an extra dose of 1200 mg eculizumab was administered and temporary corticosteroid treatment initiated. The biopsy of the right kidney revealed only chronic TMA, without active (glomerular) thrombosis or tubule-interstitial infiltrate, but with the presence of interstitial fibrosis and tubular atrophy (25%) as well as the diffuse presence of small cysts eci. Eculizumab was continued on a 6-weekly interval. Despite a temporary improvement in kidney function (eGFR 61 ml/min/1.73m^2^), an ongoing trend in CKD deterioration was observed (**Figure 2A**). Since pre-and post-renal explanatory causes could be excluded, it was suspected that the, possibly progressive, acquired glomerulocystic disease and reduced left kidney function contributed in some extent.

### Signs of TMA During Incomplete Complement Blockade

Six months later, at the age of 15 years, a sudden increase in proteinuria (UPCR 5.28 g/10mmol) and decrease in kidney function (eGFR 18 ml/min/1.73m^2^) were observed without a triggering event (**Figure 2**). Lactate dehydrogenase (LDH) was slightly elevated and thrombocytes and hemoglobin levels mildly decreased, whereas haptoglobin was normal and schistocytes were absent (**Figure 1**). In addition to these minimal signs of hematological TMA and decrease in eGFR, our patient noticed a facial edema and urticaria-like skin lesions, unrelated to eculizumab infusions, yet similar to dermatological features observed during an aHUS relapse twelve years prior. In suspicion of extra-renal TMA, skin biopsy was performed. However, only a superficial perivascular dermatitis was found, without any signs of microvascular changes. A kidney biopsy in the right kidney showed no other features than chronic TMA, similar to the previous biopsy. Complement analysis revealed signs of activation at the level of C3 (C3 1077 mg/L (700-1500), C3d 12.0 mg/L (<8.3), C3bBbP 16.8 CAU/ml (<12.0), however sC5b-9 levels were not elevated (0.50 CAU/ml). Blood pressure was elevated (129-144/78-87 mmHg), yet our patient had a variable compliance to antihypertensive medication and dietary restrictions. Since TMA could not be completely excluded, two doses of 1200 mg eculizumab were given on a weekly interval. Based on previous experience, 1200 mg of eculizumab administrations were continued on a 4-weekly interval while therapeutic eculizumab concentrations were expected. Although hematological values improved rapidly, kidney function and proteinuria only partly recovered and the gradual progression of CKD continued (**Figure 2**). Unexpectedly, low eculizumab concentrations (<8–21 µg/ml) and incomplete complement blockade (CH50 52%–122%) were observed during this 4-weekly interval period. Various factors were assessed to determine the cause of this intra-patient variability in eculizumab concentrations. Neutralizing human anti-human antibodies against eculizumab (in-house ELISA, unpublished) were not present and body weight was stable (67–70kg) during this period. C5 levels and eculizumab-C5 complexes were measured using ELISA, as described before (26, 27). Complement analysis revealed a persistent but mild elevation of C5 levels since this period, however this did not lead to an increase in eculizumab-C5 complexes (**Table 1**). In addition, sC5b-9 levels were not elevated (**Table 1**). Thorough retrospective evaluation revealed a (severe) progression in proteinuria simultaneous with the unexpectedly low eculizumab concentrations. Hence, increased urinary clearance of eculizumab was suspected. Since urine was not available for further analysis and eculizumab concentrations in our patient were only measured in plasma, we looked into the relation between eculizumab plasma concentrations and the UPCR. Empirical Bayes estimates for plasma pharmacokinetics were obtained by means of non-linear mixed effects modeling. In short, the disposition of eculizumab was described with an one-compartment model with parallel first-order and Michaelis Menten elimination. The model was based on a wide range of ages and body weights. To account for differences in pharmacokinetics between children and adults, pharmacokinetics were allometrically scaled to total body weight. Using the established model, we predicted eculizumab concentrations for our patient as well as mean population concentrations for an individual with the same weight and calculated the ratio between these concentrations. A ratio >1 implies decreased clearance of eculizumab in our patient (eculizumab concentrations are higher than expected) and a ratio <1 implies increased clearance in our patient (eculizumab concentrations are lower than expected). **Figure 2B** shows that when the UPCR increases, the ratio of predicted eculizumab concentrations is lower than 1, implying faster clearance of eculizumab, probably due to urinary eculizumab loss. In addition, concurrent with the increase in UPCR, serum IgG levels decreased (**Table 1**).

Subsequently, in attempt to rescue kidney function and rule out any level of low-grade disease activity, eculizumab interval was intensified to biweekly for four months. With this schedule, adequate CH50 (<10%) and eculizumab concentrations (>100 µg/ml) were measured. However, no improvement in kidney function was observed, and administrations were continued on a 4-weekly interval (**Figure 2A**).

### Extra-Renal TMA and Clinical aHUS Relapse

Seven months later, our patient presented with a traumatic and extremely painful ulceration. Although a skin biopsy was inconclusive for the presence of TMA, this ulcerative lesion was considered an extra-renal manifestation of TMA, especially taking into account the subsequent development of hematological signs of TMA several weeks later (**Figure 1**). In addition, kidney function and proteinuria severely worsened (eGFR 14 ml/min/1.73m^2^, UCPR 4.50 g/10mmol), and hemodialysis was started for acute on chronic kidney injury (**Figure 2**). Eventually, TMA parameters improved after switching to a biweekly eculizumab interval (eculizumab concentrations >100 µg/ml, CH50 <10%). However, further deterioration of kidney function could not be haltered and continuation of hemodialysis was required. After bilateral nephrectomy to minimize the risk for aHUS recurrence after transplantation in this complex case with unexplained, acquired, bilateral ACKD, our patient successfully received a kidney transplant from a living- related donor while on eculizumab, biweekly 1200 mg.

## Discussion

There is variation in eculizumab pharmacokinetics between individuals. Therefore, optimal, individualized therapy requires therapeutic drug monitoring and measurement of eculizumab serum levels or complement inhibition (CH50). Our case clearly demonstrates that eculizumab pharmacokinetics can vary within a patient. Initially, a 4-weekly dosing schedule resulted in complement blockade and therapeutic drug levels. However, during follow-up in a period characterized by decreasing eGFR and increasing proteinuria, a similar dosing regimen proved insufficient. To determine the cause of this intra-patient variability, all factors that could increase eculizumab clearance were evaluated.

First, neutralizing human anti-human antibodies against eculizumab or a substantial change in body weight have been previously determined to potentially influence eculizumab pharmacokinetics, but could be excluded in our patient (16). Secondly, in aHUS patients, eculizumab concentrations can be decreased by the level of C5 in serum, for example during infectious events. Normally, free eculizumab can be recycled through the neonatal Fc receptor (FcRn). One eculizumab molecule can bind up to two C5 molecules. This eculizumab-C5 complex prevents eculizumab from being recycled. Hence, elevated levels of C5 and, consequently, of eculizumab-C5 complexes can increase the apparent clearance of eculizumab by decreasing eculizumab concentrations due to reduced recycling (16). In addition to C5, eculizumab can bind sC5b-9, albeit with lower affinity. Increased levels of sC5b-9 have been shown to influence eculizumab pharmacokinetics, potentially causing lower eculizumab concentrations in serum (28). Despite a mild but persistent elevation of C5 levels after the first aHUS relapse since the initiation of eculizumab, eculizumab-C5 complexes nor sC5b-9 levels were increased during the decrease in eculizumab through levels of our patient.

In our patient, increased eculizumab clearance could be confirmed by retrospective, non-linear mixed effects modeling of eculizumab concentrations. Since other factors causing increased clearance could be excluded, the remaining explanation could be the simultaneous progression in proteinuria. UPCR reflects urinary leakage of both intermediate molecular weight (MW) proteins (e.g. albumin) and high MW proteins (e.g. immunoglobulins (IgG)) (29). Eculizumab, an IgG, is usually not excreted *via* the kidneys due to its large size. However, previous studies have confirmed the loss of functionally active IgG, including eculizumab, in urine of patients with substantial proteinuria (30, 31). In our patient, in parallel to the increase in UPCR and drug clearance, total serum IgG levels remarkably declined below the lower limit of normal. Unfortunately, urinary samples were not available to confirm the correlation between IgG (including eculizumab) leakage and our patient’s clearance.

In time, this aHUS patient developed end-stage kidney disease (ESKD) after 17-years of follow-up. One could argue that ESKD could have been delayed if the patient was treated with a biweekly eculizumab interval without therapy adjustment. However, progression of CKD started at the age of 15 years (annual eGFR decline 22.9 ml/min/1.73m^2^), directly following growth-spurt, but without any other triggering event (including an aHUS relapse). It is suspected that especially in puberty, often associated with increased deterioration of CKD, a variable compliance to medication and dietary restrictions were also not favorable to the clinical course of our patient (32). Furthermore, various other factors contributed to both the pre-existence and progression of CKD in our complex patient, including chronic (endothelium) damage due to multiple aHUS relapses during infancy and PT for over a decade, the unexplained acquired glomerulocystic disease, abnormal abdominal venous system, and reduced left kidney function.

In conclusion, we retrospectively observed a high intra-patient variability of eculizumab serum concentrations over time, probably due to an increase in urinary drug loss by proteinuria. Consequently, former eculizumab trough levels are no assurance for future pharmacokinetics or therapy effectiveness. Eculizumab serum trough levels together with complement activation (CH50) should be frequently assessed, especially in patients with elongated treatment intervals as various clinical conditions can change the eculizumab availability and, consequently, the level of complement blockade. Future studies should provide information regarding the role of proteinuria in eculizumab pharmacokinetics and urinary eculizumab monitoring in aHUS patients.

## Patient Perspective

During the whole process, the patient and his parents were informed about treatment options, risk and possibility of relapse. They were aware of the complexity of his unusual case and the patient provided written informed consent for the publication of his case.

## Data Availability Statement

The original contributions presented in the study are included in the article/supplementary materials. Further inquiries can be directed to the corresponding author.

## Ethics Statement

Written informed consent was obtained from the individual(s) for the publication of any potentially identifiable images or data included in this article.

## Author Contributions

Research idea and study design: RB, MT, RT, EV, NV. Data analysis/interpretation: RB, MT. Supervision: NV, EV, RT, JW, LV. Manuscript drafting: RB, KW, NK. Manuscript reviewing: MT, CD, EV, RT, JW, LV. All authors contributed to the article and approved the submitted version.

## Conflict of Interest

JFW is a member of the international advisory board of Alexion and also received a grant from Alexion.

The remaining authors declare that the research was conducted in the absence of any commercial or financial relationships that could be construed as a potential conflict of interest.
